# Normal aging in human lumbar discs: An ultrastructural comparison

**DOI:** 10.1371/journal.pone.0218121

**Published:** 2019-06-20

**Authors:** Ricardo B. V. Fontes, Josemberg S. Baptista, Said R. Rabbani, Vincent C. Traynelis, Edson A. Liberti

**Affiliations:** 1 Department of Neurosurgery, Rush University Medical Center, Chicago, Illinois, United States of America; 2 Laboratory of Applied Morphology - LEMA, Universidade Federal do Espirito Santo, Vitoria, Espírito Santo, Brazil; 3 Department of General Physics, Instituto de Fisica, Universidade de Sao Paulo, Sao Paulo, São Paulo, Brazil; 4 Department of Anatomy, Instituto de Ciencias Biomedicas, Universidade de Sao Paulo, Sao Paulo, São Paulo, Brazil; Pennsylvania State Hershey College of Medicine, UNITED STATES

## Abstract

The normal aging of the extracellular matrix and collagen content of the human lumbar intervertebral disc (IVD) remains relatively unknown despite vast amounts of basic science research, partly because of the use of inadequate surrogates for a truly normal, human IVD. Our objective in this study was to describe and compare the morphology and ultrastructure of lumbar IVDs in 2 groups of young (G1—<35 years) and elderly (G2—>65 years). Thirty L4-5 and L5-S1 discs per group were obtained during autopsies of presumably-asymptomatic individuals and analyzed with magnetic resonance imaging (MRI), a morphological grading scale, light microscopy, scanning electron microscopy (SEM) and immunohistochemistry (IHC) for collagen types I, II, III, IV, V, VI, IX and X. As expected, a mild to moderate degree of degeneration was present in G1 discs and significantly more advanced in G2. The extracellular matrix of G2 discs was significantly more compact with an increase of cartilaginous features such as large chondrocyte clusters. Elastic fibers were abundant in G1 specimens and their presence correlated more with age than with degeneration grade, being very rare in G2. SEM demonstrated persistence of basic structural characteristics such as denser lamellae with Sharpey-type insertions into the endplates despite advanced age or degeneration grades. Immunohistochemistry revealed type II collagen to be the most abundant type followed by collagen IV. All collagen types were detected in every disc sector except for type X collagen. Statistical analysis demonstrated a general decrease in collagen expression from G1 to G2 with an annular- and another nuclear-specific pattern. These results suggest modifications of IVD morphology do not differ between the anterior or posterior annulus but are more advanced or happen earlier in the posterior areas of the disc. This study finally describes the process of extracellular matrix modification during disc degeneration in an unselected, general population and demonstrates it is similar to the same process in the cervical spine as published previously.

## Introduction

Pathologic conditions of the spine have been described ever since medical knowledge was placed in written form. The management of fractures and dislocations is featured in the Edwin Smith papyrus (c. 1600 BC) and Hippocratic writings (c. 400 BC) [1,2]. Low back pain due to degenerative conditions, however, would only feature prominently in medical texts during the Industrial Age, possibly due to a combination of an increase in life expectancy, technical advances in surgery and even the emergence of litigation for work- and accident-related health issues[3]. Today the lifetime prevalence of low back pain (LBP) approaches 80% and it is not only one of the most common general medical complaints but is the biggest cause of years lived with a disability [4–6]. Accordingly, a great body of literature has been produced since the mid-19^th^ century focusing on the anatomy and degeneration of the intervertebral disc (IVD). Its basic structure has been described since at least 1858 and a very important series of three papers on modifications induced by aging was published in 1945[7,8].

Despite the large amount of data produced, relatively little is known about the normal aging of the extracellular matrix (ECM) and collagen fiber system of the human intervertebral disc (IVD). Most of the morphological data regarding ECM and collagen was produced in discs from other animal species with markedly different structure or obtained during surgery for different diseases such as scoliosis or LBP and thus cannot be considered “normal” discs[9–11]. Other biases such as unknown spinal segment or the sole utilization of qualitative methods are also frequent [12]. We sought to address this knowledge gap in ECM and collagen modification during normal aging by performing a multimodal comparison of lumbar discs from likely asymptomatic young (under 35 years) and elderly (over 65 years) individuals, with the hypothesis that although structure is generally unchanged, noticeable differences are evident at the ultrastructural level.

## Material and methods

This work was performed with human whole L4-5 and L5-S1 discs obtained during unselected autopsies as described in our previous study with cervical discs [13]. The larger size of lumbar discs and the usual presence of an identifiable nucleus pulposus (NP) allowed them to be divided into 3 sectors for analysis: *anterior* (aAF), a middle fragment (NP) and *posterior* (pAF). This study was granted IRB approval at ICB-USP. Briefly, thirty L4-S1 vertebral blocks were collected from unselected autopsies of recently-deceased (<6 hours) cadavers at the SVOC-USP. Next of kin provided written consent and were interviewed to exclude individuals with known history of neck or back pain, neoplasms or rheumatological conditions[14]. Specimen age and data is provided in Table 1. Group 1 (**G1**) was comprised of 15 young cadavers (<35 years) and group 2 (**G2**) included 15 specimens from cadavers aged 65 or older. Throughout the study, L4-5 and L5-S1 discs were analyzed jointly, thus resulting in 30 discs/age group. Specimens were assigned random identifiers and masked to researchers.

**Table 1:** Cadaver data.

	G1^a^	G2^a^	*p*^b^
Age (yrs)	31.8 +/- 2.6	78.1 +/- 7.8	<0.001
Height (cm)	172.6 +/- 8.0	166.0 +/- 9.4	0.07
Weight (kg)	72.5 +/- 14.7	68.4 +/- 22.0	0.06
Male:Female	16:4	13:7	—

^a^Average +/- standard deviation.

^b^*p*, Student’s T analysis of **G1** versus **G2**.

Multimodality structural and ultrastructural comparison was performed as described in our previous work[13]:

### MR imaging

Ten discs per group were submitted to 1.5T MR imaging with 2mm mid-sagittal and axial cuts through the level of the L4-5 and L5-S1 discs. MR parameters were adapted to replicate a T2 sequence in our specimens (matrix = 512x225, TR/TE 5000/130ms and FOV = 140x140). Discs were analyzed semi-quantitatively with a modified Okada scale from 0 to 6 and compared with the Mann-Whitney test (GraphPad Prism 6, San Diego, CA)[13]. Level of significance of .05 was utilized throughout the study.

### Morphological grading

Following fixation in 4% formaldehyde for 6 months, all specimens were sectioned in the mid-sagittal plane and graded semi-qualitatively with the Thompson scale (1 to 5) and compared with the Mann-Whitney test.

### Light microscopy

Discs and their endplates were decalcified in 0.25M EDTA for 30 days and 1M for 5 days immediately before processing. aAF, NP and pAF fragments were frozen-sectioned on a sagittal (20 discs/group) or coronal (10 discs/group) orientation. Semi-serial, 8μm sections were stained with hematoxylin-eosin (HE), Sirius Red (SR), Verhoeff’s iron-hematoxylin (mature elastic fibers) and Weigert’s resorcin-fuchsin (elastic and elaunin fibers) techniques[15]. Photomicrographs were acquired under normal and polarized (Sirius Red) light.

### Scanning electron microscopy (SEM)

Six discs from **G1** and **G2** each were randomly selected for SEM. The clean-cut mid-point surface of each specimen was attached face-up to an SEM stub[16]. The aAF, NP and pAF lumbar fragments, from each disc, were dehydrated (45°C for 12 hours), gold-coated and analyzed in a scanning electron microscope (Leo 435 VP, Cambridge, England).

### Collagen immunohistochemistry (IHC)

Six discs from **G1** and **G2** each were randomly selected. A commercially-available ABC kit was utilized (ImmunoCruz ABC, SantaCruz Biotechnology, California). 8 μm-thick sections of each sector (aAF, NP and pAF) were prepared according to manufacturer instructions—protease-based antigen unmasking (Table 2—30 minutes, 37°C), neutralization of endogenous peroxidase (1% H_2_O_2_ in PBS, 5 minutes), blockage of non-specific sites (1.5% blocking serum, 30 minutes), incubation with primary (12 hours, 4° C) and secondary antibodies (30 minutes, 37° C) and avidin-peroxidase conjugate (30 minutes, 25° C). DAB chromogen was allowed to react for 3 minutes and slides assembled[12]. Each step was preceded by two PBS washes. Primary antibodies against human collagen types I, II, III, IV, V, VI, IX and X were utilized with single- (no primary) and double-negative (no antibody) controls. Quantification was performed with area-based method and expressed as a percentage as previously reported[13,17]. Ten random fields at 1000x magnification were sampled and expression of each collagen compared to the negative controls with one-way ANOVA and *post-hoc* Tukey tests to determine if different from background or non-specific binding. Comparisons between disc regions and groups were performed with the T test without adjustment for multiple comparisons as described[13,18].

**Table 2:** Antibodies and proteases utilized in the study.

Antigen	Protease	Antibody data
Collagen I	0.2% trypsin	Abcam, ab90395
Collagen II	0.4% pepsin in 0.01N HCl	Santa Cruz, sc-59958
Collagen III	0.4% pepsin in 0.01N HCl	Sigma-Aldrich, C7805
Collagen IV	0.4% pepsin in 0.01N HCl	Sigma-Aldrich, C1926
Collagen V	0.4% pepsin in 0.01N HCl	Millipore, MAB3393
Collagen VI	0.4% pepsin in 0.01N HCl	Santa Cruz, sc-47712
Collagen IX	0.4% pepsin in 0.01N HCl	Millipore, MAB3304
Collagen X	0.4% pepsin in 0.01N HCl	Sigma-Aldrich, C7574

## Results

### Magnetic resonance imaging

**Fig 1:**
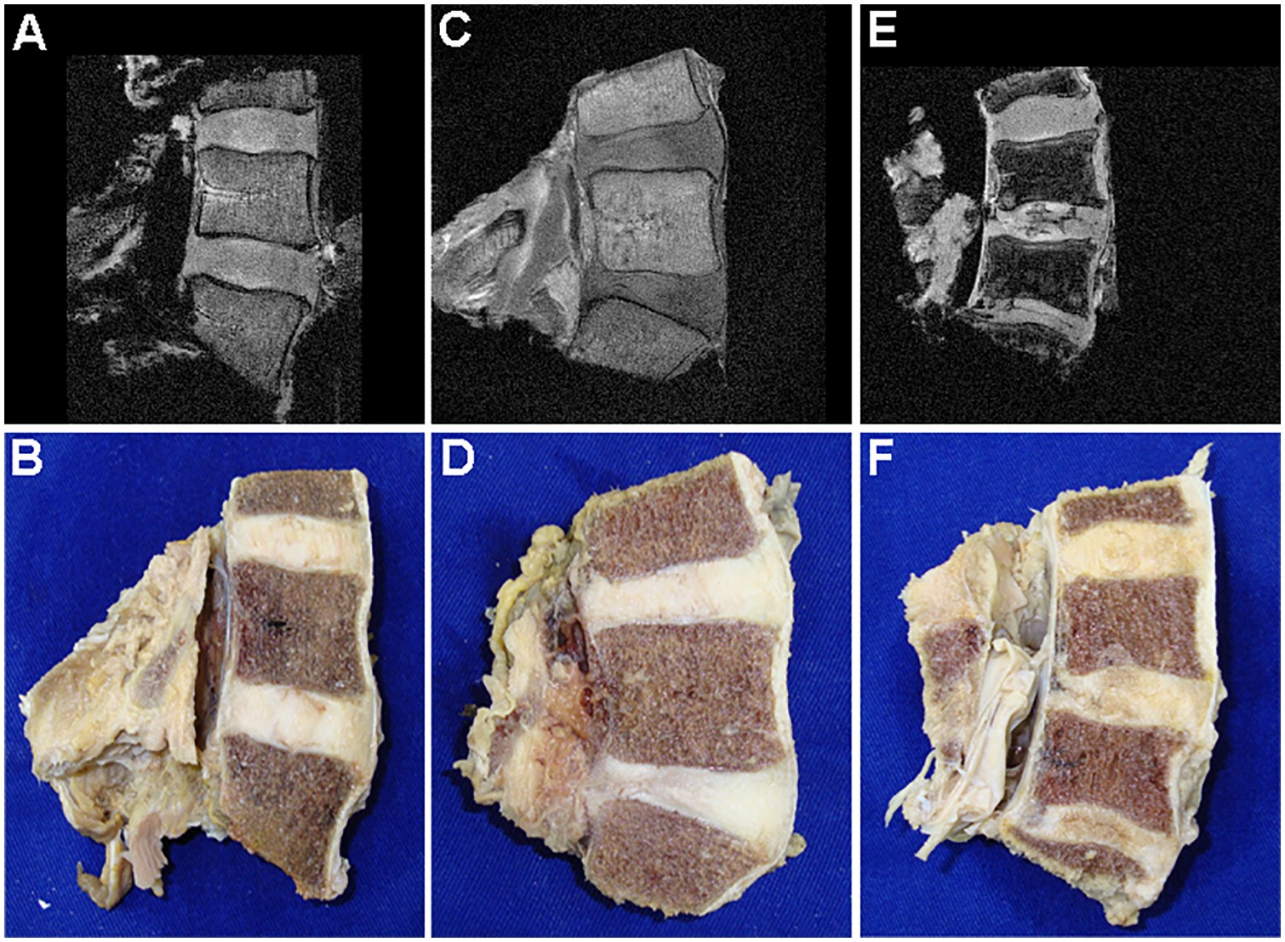
MR and corresponding mid-sagittal views of lumbar vertebral blocks. A typical **G1** specimen (**A**, **B**) is shown with incipient degenerative findings. Advanced degenerative findings in **G1** specimens were almost always posterior annular tears as shown in the L5-S1 disc of this other specimen (**C**, **D**–Okada 3, Thompson 4 for L5-S1). Forty percent of all **G2** discs exhibited structural failure as shown in **E** and **F** (Okada 6 and Thompson 5).

**Fig 2:**
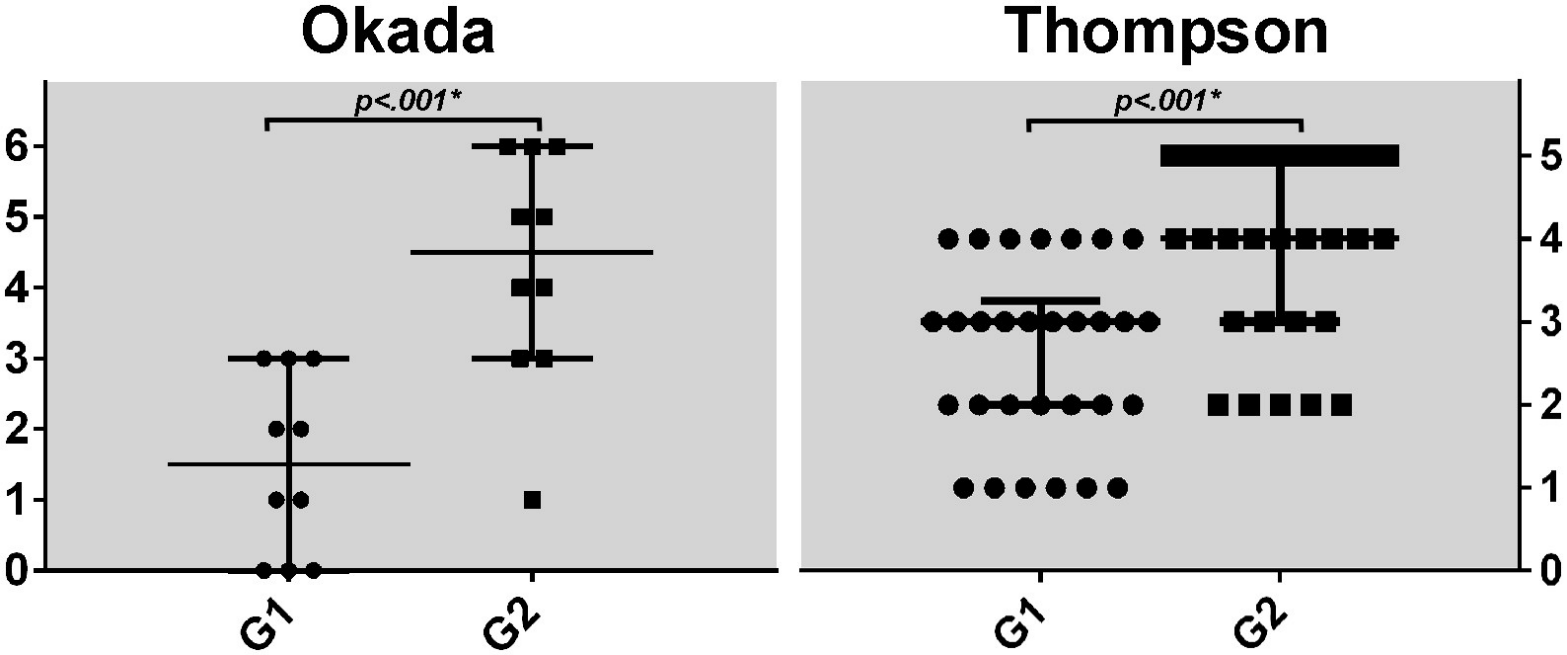
Distribution of Okada (n = 10) and Thompson (n = 30) scores per group. Bars, median and interquartile range. Mann-Whitney test.

Mild and moderately-advanced degenerative findings were common in **G1** cadavers (Fig 1). While most specimens had preserved height and no to minimal adjacent osteophytes, the AF-NP separation was frequently blunted. Half of the studied discs had low (0–1) Okada scores. MRI could on occasion detect horizontal tears in the posterior aspect of the disc such as in Fig 1C but no example of complete ankylosis was seen in **G1**. Degenerative alterations were far more evident in **G2** specimens. MR could demonstrate osteophytes, large tears and ankylosis effectively and thus Okada scores were significantly higher (*p* < .001) than in **G1** (Fig 2).

### Morphological grading

When comparing MR and morphological results, small disc tears were usually not visualized under the utilized MR settings. Discs with little to no degeneration (Th1-2) were common in **G1** (13/30) but infrequent in **G2** (5/30, all Th2). On the other end of the degenerative spectrum, advanced findings were present in 7 of the 30 **G1** discs (all Th4) and predominant in **G2** (9/30 Th4 and 12/30 Th5). Thompson 4 discs in **G1** usually received that grade due to small AF tears being present. Every degenerative feature described in the modified Thompson criteria were represented in **G2** –disc collapse, osteophytes, loss of AF-NP distinction, focal sclerosis and horizontal fissures (Fig 1F). Variability within the lumbar segment was small with only one cadaver in **G1** and two in **G2** demonstrating a Thompson difference of 2 grades, and none of 3 grades. Accumulation of degenerative features from **G1** to **G2** was also confirmed statistically (*p* < .001 –Fig 2).

### Light microscopy

**Fig 3:**
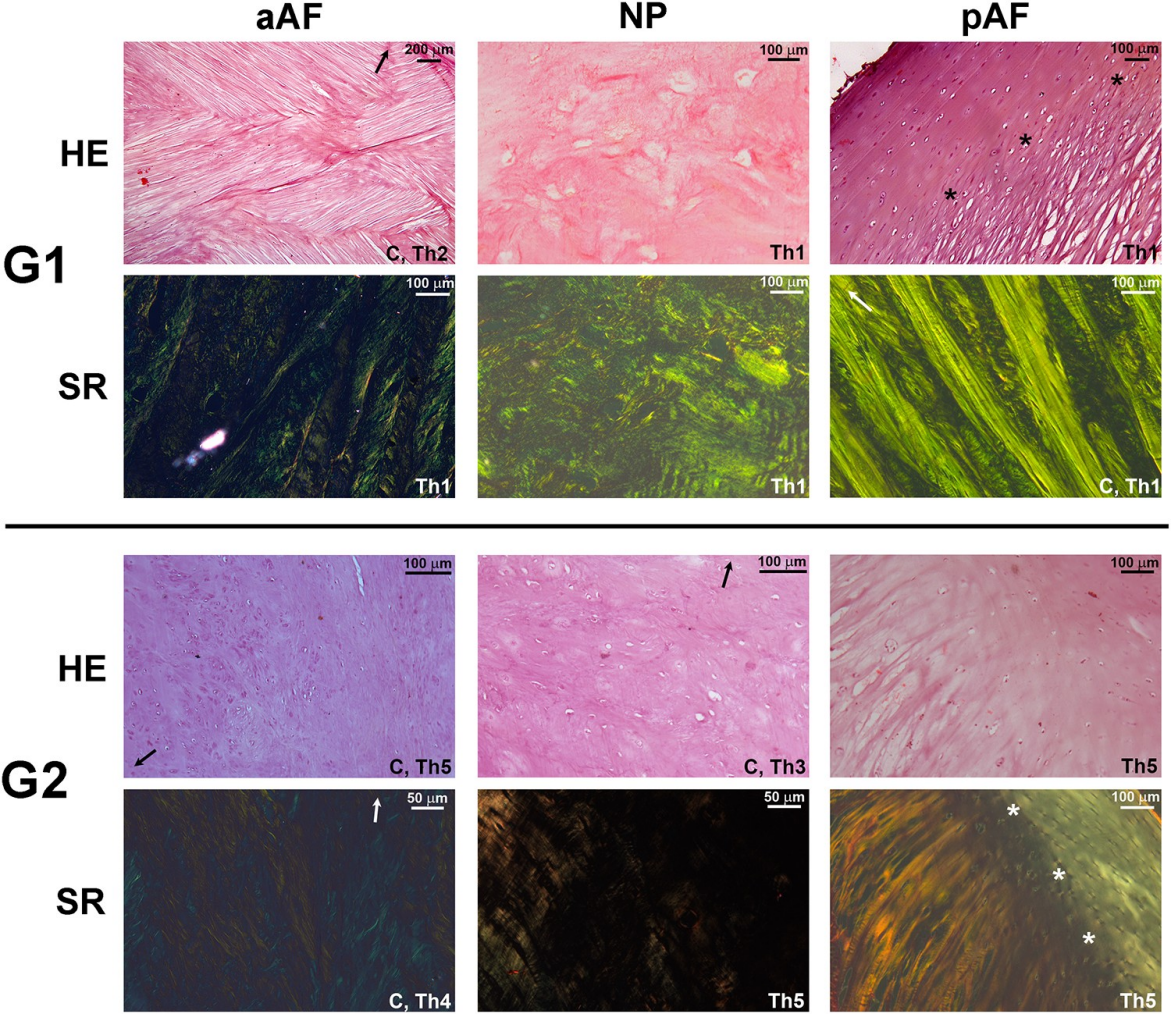
Light microscopy images. Alternating diagonal and longitudinal lamellae are visible in the aAF and predominantly longitudinal in the pAF. Sharpey-type insertion of these fibers into the endplate is visualized in **G1** (asterisks) and **G2**. The loose fibrocartilaginous phenotype of the **G1** AF is substituted for a cartilaginous one in **G2** with dense extracellular matrix and chondrocyte clusters. HE, hematoxylin & eosin. SR, Sirius Red. “C” designates a coronally-oriented section, Th notes Thompson grade, arrow indicates the nearest endplate.

The AF consisted of alternating lamellae of longitudinal and diagonal orientation; nowhere in the AF was a purely circular fiber orientation found (Fig 3). These lamellae were not clearly distinct from the anterior (ALL) and posterior (PLL) longitudinal ligaments and possessed a strong insertion into the vertebral endplate similar to Sharpey’s fibers. pAF fibers had a predominantly longitudinal orientation with few diagonal lamellae. The NP consisted of loose connective tissue without a particular orientation. SR-stained sections under polarized light demonstrated a prevalence of yellow and green refringence in the AF with dark background in the NP. The overall aspect of the **G1** “young” phenotype was that of well-arranged fibrocartilaginous lamellae in the AF separated by 1–2μm and few chondrocytes, either in isolation or 2–3 cell clusters. **G2** specimens demonstrated a loss of the fibrous component and had a predominantly cartilaginous phenotype, with substitution of the lamellar arrangement in the AF and loose extracellular matrix of the NP for a denser matrix with frequent and large chondrocyte clusters, proliferating into the disc from the adjacent vertebral endplates (Fig 3). Polarization also demonstrated a darker background throughout **G2** discs with a smaller component of red refringence.

**Fig 4:**
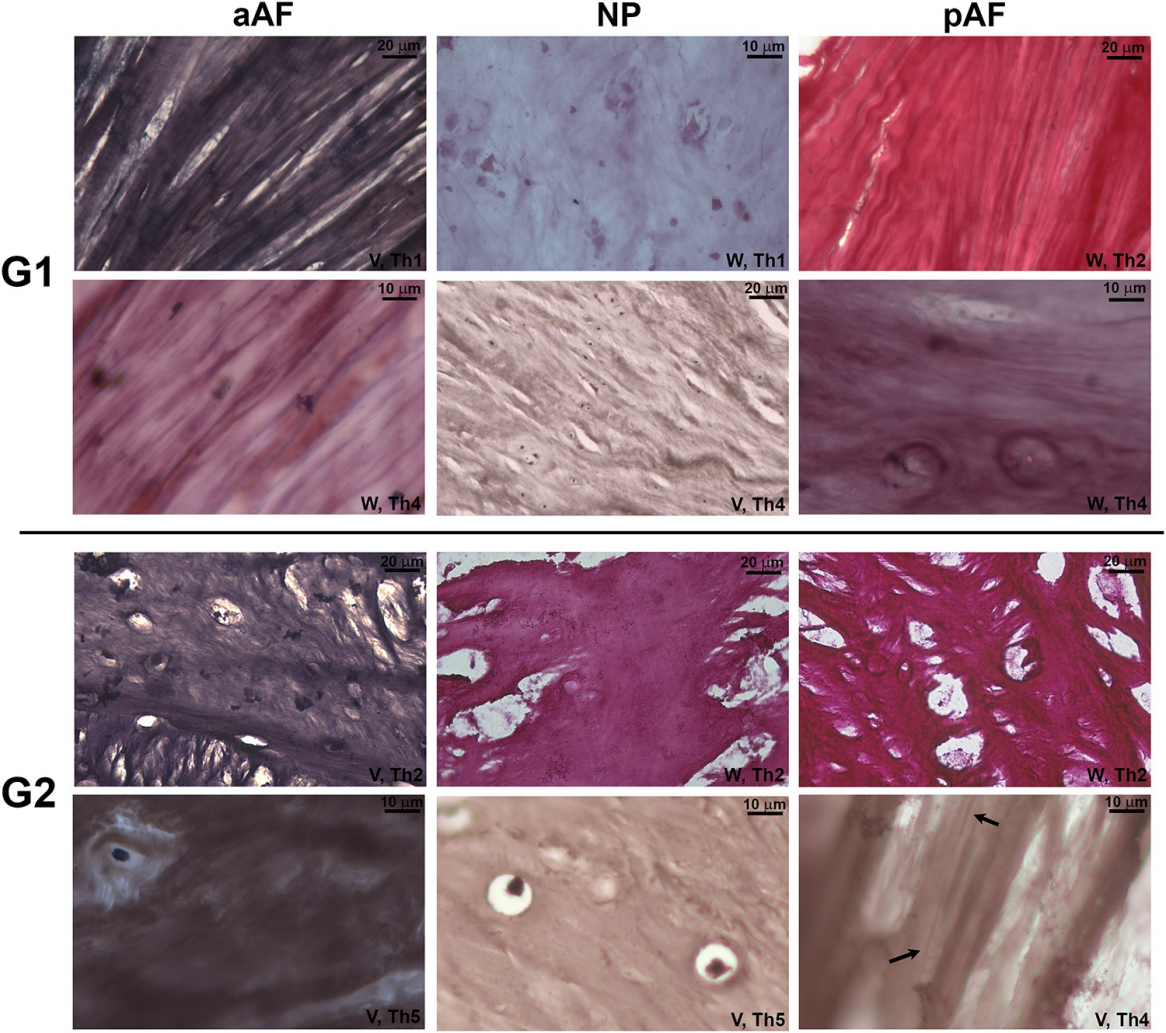
Elastic fiber stains in G1 and G2 lumbar discs. The presence of elastic fibers was more related to group (age) than to degeneration grade; even Th4-5 **G1** specimens had prominent fibers in the AF while mildly-degenerated **G2** discs (Th2) uniformly lacked them. The single **G2** specimen is shown with few elastic fibers in the pAF (arrows). V, Verhoeff; W, Weigert; Th1-5, Thompson grade.

The presence of elastic fibers in Verhoeff- and Weigert-stained slides was congruent, i.e., suggests these fibers are of the mature elastic type (Fig 4). These were predominantly found in the AF of **G1** specimens aligned within the connective tissue bundles. Elastic fibers were occasionally seen in the **G1** NP but did not exhibit any particular alignment (Fig 4, **G1** NP). The presence or absence of elastic fibers seemed to correlate more to age than to degree of degeneration as measured by the Thompson scale–Fig 4 lists examples of **G1** Th4 discs with abundance of elastic fibers and **G2** Th2 specimens lacking them.

### Scanning electron microscopy

**Fig 5:**
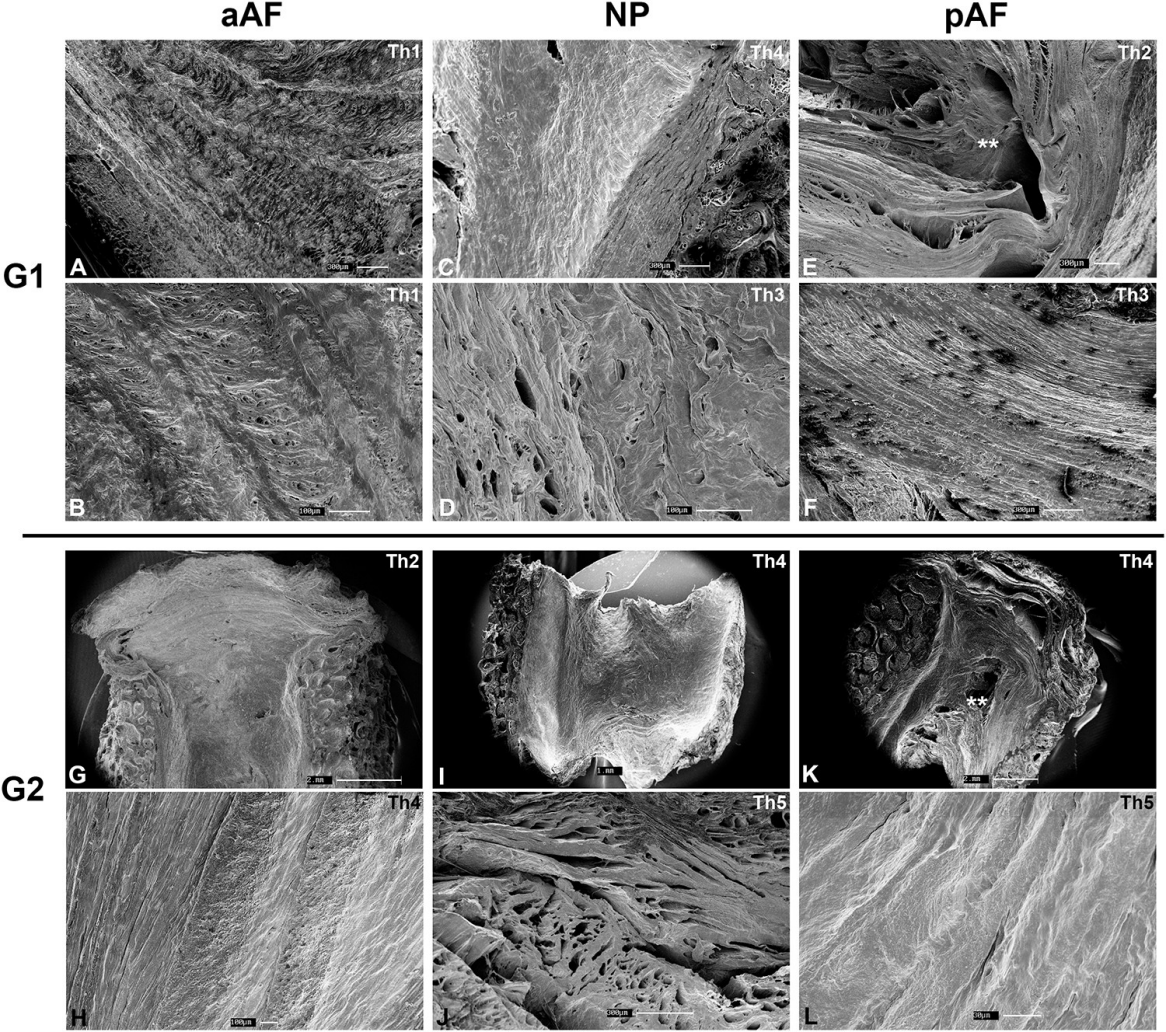
Scanning electron microscopy. Typical AF arrangement seen in **5A**, with 1–2μm interlamellar space. Endplate insertion of AF fibers is visible in **5G** and **5K** and not present in the NP (**5C** and **5I**). A cartilaginous phenotype predominates in **G2** specimens, with a denser matrix in both the AF and NP. Lamellae are compact and no space is visualized even at higher magnification (**5H** and **5L**). pAF tears are demonstrated (**) in **G1** (**5E**) and a **G2** (**5K**).

The morphological aspects of the lumbar discs described above were further demonstrated from a tridimensional perspective with cut-surface SEM. The lack of ALL and PLL separation from the superficial layers of the AF (Fig 5A, 5G and 5K), the lamellar arrangement of the AF (Fig 5A, 5B and 5H), a clearly-distinguishable NP, the Sharpey-type insertion of AF fibers into the endplates (Fig 5G and 5K) and its absence in the NP (Fig 5C and 5I) are general features maintained at the ultrastructural level in both **G1** and **G2**, regardless of the macroscopic grading of degeneration. Denser AF and NP matrix with disappearance of the interlamellar space is particularly evident under SEM and higher magnification in **G2** (Fig 5H and 5L).

### Collagen immunohistochemistry

**Fig 6:**
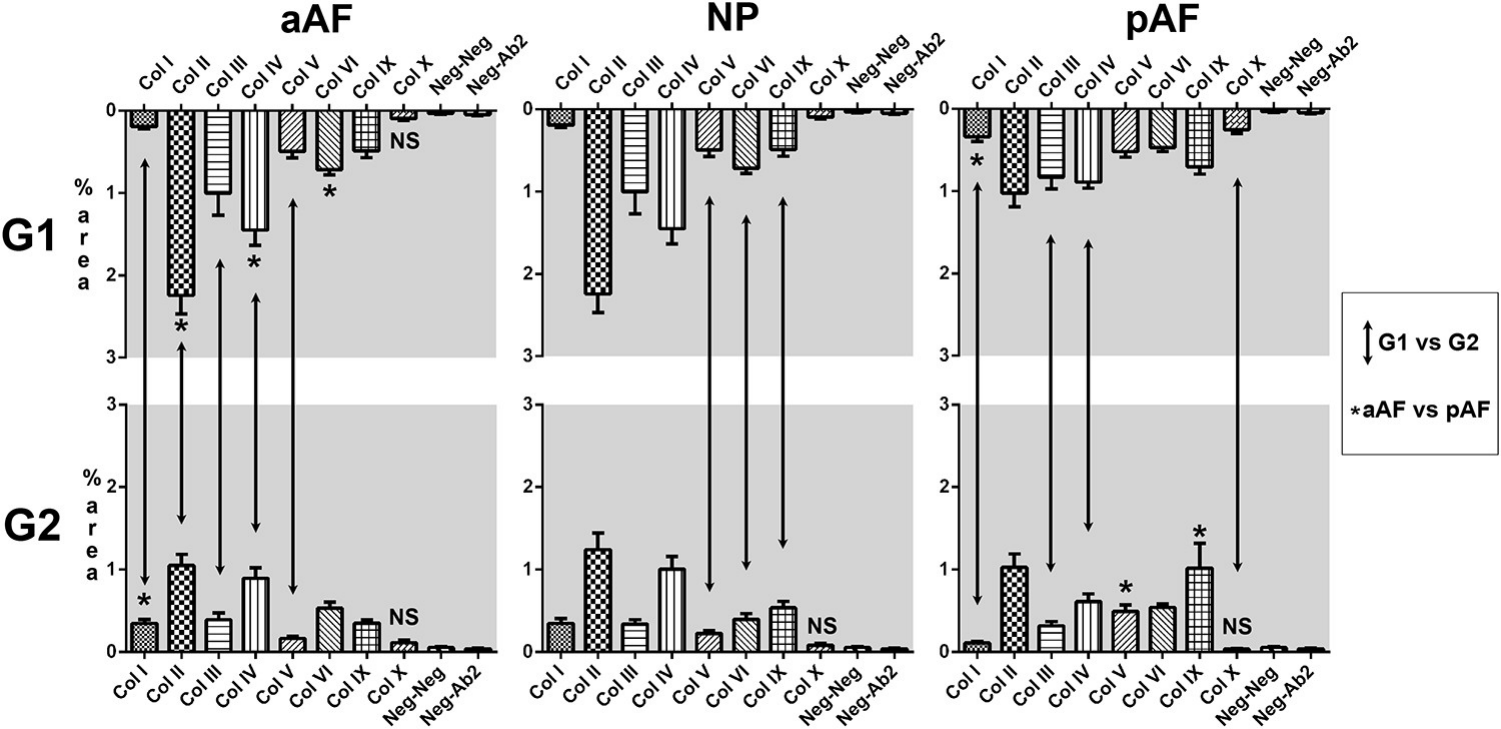
Stained area (%) per collagen type. Vertical arrows indicate *p* < .05 in **G1** vs. **G2** comparison. Black asterisks indicate *p* < .05 in the aAF vs. pAF comparison and are located in the frame with higher collagen reactivity. NS, not significant. Error bars, standard error of the mean.

The collagen expression was found to be significantly higher than non-specific antibody binding or background for all collagens in every lumbar disc sector except for collagen X, which was only encountered in the **G1** NP and pAF (Fig 6). Qualitatively, two main staining patterns were observed: types I, II, IV and IX exhibited filiform, extracellular expression while types III, V and VI stained in an intra- and pericellular pattern. Area-based quantification of collagen expression and the statistical comparisons performed (**G1** vs. **G2**, aAF vs. pAF) enabled three general observations to be made: collagen II and IV are the main structural types in lumbar discs, there is a general decrease in collagen expression from **G1** to **G2** and collagen expression pattern is similar across disc sectors but preferentially higher in the anterior disc region. Out of 66 statistical analyses performed, 27 resulted with *p* < .05 and only 4 of these 27 constituted exceptions to the three observations above: collagen I in the aAF (pAF higher than aAF and increases from **G1** to **G2**), collagen V in **G2** pAF (pAF higher than aAF) and collagen IX in **G2** pAF (pAF higher than aAF). The **G1** vs. **G2** comparison demonstrated different aging patterns in the AF and NP: AF degeneration was marked by reductions in the expression of collagens II, III and IV while degeneration in the NP was characterized by a decrease in the expression of collagens V, VI and IX.

## Discussion

There is a wealth of basic science data on lumbar disc degeneration produced since Schmorl and Junghanns’ pioneering study in 1932 but how much of it is directly and perfectly applicable to human disc degeneration is unknown. For example, discs from quadruped species, particularly canines, have a propensity to undergo calcification during normal degeneration, while the rat tail suspension model has been extensively demonstrated to differ from normal human disc aging from both morphological and molecular standpoints[10,11,19]. Lumbar discs from patients undergoing deformity correction surgery have different mechanical and matrix properties than those undergoing surgery for degenerative disc disease, including different collagen and calcium content and are inadequate surrogates for normal or even pathological degeneration[9,20]. Collagen content in discs from other animal species exhibit variations in collagen composition ranging from 50 to 600% of the equivalent human content, despite prior interpretations of this collagen content being “similar”[21]. *In vitro* and animal models are thus very useful for testing therapeutic possibilities in a pre-clinical setting but are inherently poor choices to understand ECM modifications during normal disc aging, while discs from human patients being treated for other pathologies should be reserved for the study of those particular conditions[22].

That degenerative alterations of the lumbar IVD may be present and even progress while remaining asymptomatic is well-known to spine surgeons in both young and elderly individuals[23,24]. Degeneration occurring simultaneously in the cervical and lumbar segments has also been demonstrated in almost 80% of asymptomatic elderly subjects[25]. Our results suggest this number is close to 100% and lower figures probably reflect a limitation of the imaging method (MRI) utilized—even at 1.5T, morphological grading in our series tended to be slightly worse than MR grading likely due to the inability to detect and account for small disc fissures. As MRI technology continues to improve and become more available, the incidence of degenerative findings is likely to increase and additional features can be incorporated into proper, segment-specific grading schemes [26].

The morphological arrangement of the fibrocartilaginous young AF is easier to appreciate in lumbar specimens due to their larger size. Contrary to most textbook descriptions of a circular pattern, lamellar arrangement was predominantly diagonal and longitudinal, with a predominance of the latter in the pAF. An example of alternating diagonal lamellae is seen in Fig 3, G1 aAF HE stain, whereas the longitudinal pattern is seen in the same Fig 3, G1 pAF SR stain. Endplate insertion of AF fibers had been first noted by Coventry *et al*. in 1945 but over the years it has remained under debate whether these insertions are superficial in subchondral bone only or reach subchondral bone as real Sharpey fibers [27–29]. SEM results show both theories are correct and depend on location within the disc–Fig 5G and 5K show that more superficial fibers next to the ALL and PLL subchondral but the majority of AF insertions are restricted to cartilage. This insertion is thought to confer additional resistance to shearing and rotational stresses[27]. Our results also demonstrate the absence of these insertions in the NP and is not surprising given the distinct embryological origins[30,31].

The presence of elastic fibers in the lumbar segment seemed to correlate more with age than with the morphological grading of degeneration (Fig 4), usually in higher density in the AF than in the NP within each age group. However, the lack of elastic fibers in even Th2 **G2** specimens is surprising and may reconcile different views on the behavior of elastic fibers during normal aging. Their detection in human discs had long been elusive until Buckwalter *et al*. demonstrated their presence with transmission electron microscopy[32,33]. While unknown disc origin may have influenced several studies, in 2007 Cloyd and Elliott reported that elastin content actually increased with disc degeneration, which would apparently contradict data from studies involving other joints[34,35]. These authors had already suggested that the answer might lie with patient age, a factor not accounted for in their study and Fig 4 clearly demonstrates how a young disc may receive an elevated degenerative grading due to an annular tear and still maintain its elastic fibers due to its ECM not having undergone cartilaginous modification[34].

Based on the predominance of yellow and red refringence in the polarized light analysis, we expected a significantly higher content of collagen I. However, in all analyzed situations reactivity to collagen II stained a larger area. Our group relied heavily on SR polarization in the past to analyze large areas with a fraction of the labor required to perform IHC but this mismatch suggests SR refringence may not be specific enough. The same mismatch had been reported before in the IVD by Nosikova *et al*. and the cause may lie in the physical principle for generation of refringence in SR staining, which relies on the different fiber length and diameter of each collagen subtype. In compact structures with an elevated collagen content, a varying fiber alignment can introduce bias when the structure is sectioned and IHC should be a preferred method[15,27,36,37].

Similarly to cervical IVDs, collagen II and IV were present in a greater area than any other subtype, with the collagen IX **G2** pAF exception noted above[13]. The expression of collagen IX in this particular site, however, had the largest variation of all measurements performed and may be an artifact. The reduction in collagen II expression with aging was the most consistent of all results but its expression was already low in the young **G1** pAF did not change significantly from **G1** to **G2**. The variations in collagen expression thus suggest that the completely senescent, “end-degeneration” profile is similar across different disc regions and segments but is more advanced temporally in the lumbar segment and posterior disc regions: it is likely that even at 35 years of age the ECM of the pAF was already modified with a lower collagen II expression. A similar ultrastructure and collagen profile in IVDs across different vertebral segments had been shown before in *Macaca fascicularis* but not in humans[38]. Nerlich *et al*. had suggested a slightly different pattern of collagen substitution—for example, an increase in collagen I with aging and that collagen IV would be a marker of early degeneration but not a normal constituent of the disc—but the main feature of a decrease in collagen II was also seen. Comparison is ultimately difficult because of semi-qualitative grading of collagen expression in their study [12].

One of the limitations of this study is that the deceased individual might have been symptomatic, unknown to the interviewed family members, a deficiency it shares with our cervical study[13]. At first glance, this design may have resulted in a seemingly small number of subjects but we believe this is balanced by the inclusion of sufficient data points in the collagen analysis (60 per type and sector) and rigorous statistical analysis. It is still the largest study of this type involving the labor-intensive methods of SEM and IHC. Short of prospectively enrolling a large population of human subjects, we see no alternative to address the matter of unknown symptoms. We thus interpret these results as applicable to a general, unselected, presumably-asymptomatic population and demonstrates that our hypothesis is in fact true—there is an NP- and an AF-specific pattern of ECM modification during aging. It is also very similar in the cervical spine[13].

These results are particularly relevant for disc regenerative (e.g., stem cell-based) or replacement (e.g., tissue-engineered prostheses) therapies. The simple observation that the degenerative process is similar across the spine should obviate the need for segment- or region-specific therapies, greatly facilitating this process. We feel our results have succeeded in providing a morphological and ultrastructural “roadmap” of disc and especially ECM degeneration that can be potentially utilized to guide or monitor such therapies.
